# Einthoven dissertation prizes 2012

**DOI:** 10.1007/s12471-013-0404-0

**Published:** 2013-04-18

**Authors:** E. E. van der Wall, W. H. van Gilst, M. J. Schalij, V. Umans

**Affiliations:** 1 Interuniversity Cardiology Institute of the Netherlands (ICIN)—Netherlands Heart Institute (NHI), Utrecht, the Netherlands; 2 Department of Cardiology, Leiden University Medical Center, Leiden, the Netherlands; 3 Department of Cardiology, Medical Center Alkmaar, Alkmaar, the Netherlands

For the 24th time in a row the Interuniversity Cardiology Institute of the Netherlands (ICIN-Netherlands Heart Institute) and the Netherlands Society of Cardiology (NVVC) supported the competition for the best three cardiovascular PhD theses published in the year 2012. The dissertation prize carries the name of one of the greatest Dutchmen in the history of cardiovascular medicine, Willem Einthoven, who in 1902 for the first time recorded the human ECG, for which he received the Nobel Prize in 1924.

This time the jury received a total of 34 PhD dissertations. The jury members were very much impressed by the high scientific quality of the PhD fellows. Based on scientific originality, the number of articles in first-rate journals, the contribution as a first author (or shared first author) to the papers, and the number of citations, the jury finally selected three nominees: Dr. A.S. Amin, (Academic Medical Centre Amsterdam), Dr. R.J. van Bommel, (Leiden University Medical Center), and Dr. R Pisters (Maastricht University Medical Centre).

The members of the jury were: M. Alings, H.J. Crijns, J. Deckers, W.H. van Gilst, V. Umans, and E.E. van der Wall.

The three candidates presented their PhD thesis at the annual Spring Congress of the NVVC at the Congress Centre De Leeuwenhorst, in Noordwijkerhout, on 4 April 2013. The ultimate winners of the first, second and third prize were chosen by the audience. Summaries of the three nominated PhD theses are given below.

**Modifiers of phenotype in inheritable arrhythmia syndromes**

**Fig. 1:**
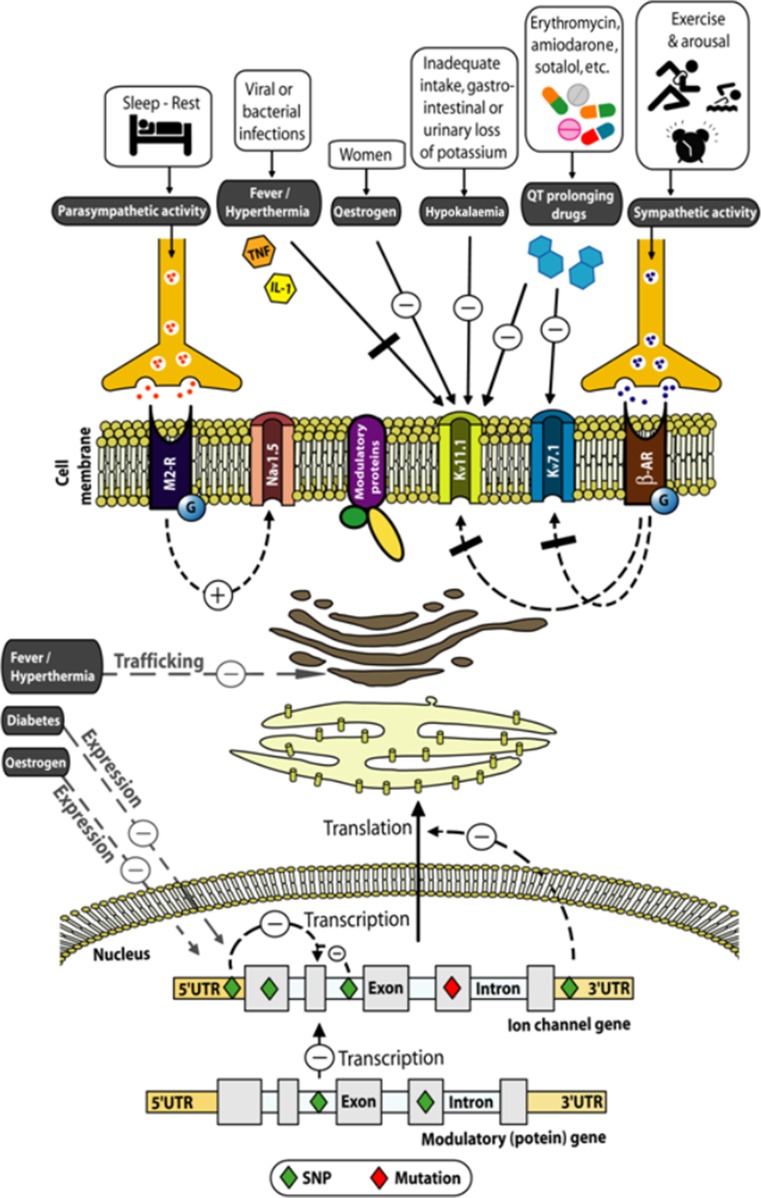
Genetic and environmental factors that potentially modify the phenotype in inherited arrhythmia syndromes

Single autosomal-dominant mutations in genes encoding for cardiac ion channels serve as the primary genetic substrate for inheritable arrhythmia syndromes such as the long-QT syndrome (LQTS) and the Brugada syndrome (BrS) [1]. This discovery has been the basis for staggering numbers of subjects and families counselled worldwide for inheritable arrhythmia syndromes and (prophylactically) treated based on finding a mutation [2]. However, therapy is greatly hampered by the growing awareness that simple carriership of a mutation often fails to predict phenotype: many mutation carriers never develop clinically relevant disease while others experience sudden cardiac death at a young age. It is still largely elusive what determines this variability in disease severity, where even family members with an identical mutation still display large differences in disease severity [1,3]. This suggests that additional factors modify the phenotypic manifestations of a disease-causing mutation. The research described in this thesis aimed to uncover potential genetic and environmental factors that, in conjunction with a mutation, modify the phenotype in inherited arrhythmia syndromes (Fig. 1).

*Fever and exercise as modifiers of phenotype*

Our clinical studies demonstrate that in patients with BrS, linked to mutations in *SCN5A* (the gene encoding for the cardiac sodium channel), fever increases the risk of cardiac arrest due to ventricular tachycardia/fibrillation [4], and fever and exercise, separately, aggravate the typical BrS ECG changes (i.e., more PR and QRS widening and further right-precordial ST segment elevation) [4–5]. Moreover, our data suggest that timely use of antipyretics reduces the risk for cardiac arrest during fever. Our experimental studies demonstrate that BrS-linked *SCN5A* mutations impair cardiac sodium channel function more severely at higher body temperatures and at faster heart rates, thereby providing a molecular mechanism for the phenotype-modifying role of fever and exercise in patients with BrS [6–7]. We also provide clinical and experimental evidence that fever prolongs QTc duration and triggers torsade de pointes ventricular tachycardia and ventricular fibrillation in some patients with LQTS type 2, the second most prevalent type of LQTS [8].

*Single nucleotide polymorphisms as modifiers of phenotype*

We show that single nucleotide polymorphisms (SNPs) in the 3′untranslated region (3′UTR) of *KCNQ1*, the gene encoding for a major cardiac potassium channel, modify the phenotype in LQTS type 1, the most prevalent type of LQTS [2]. MicroRNAs bind to the 3′UTR and thereby inhibit the translation of the messenger RNA to protein [9]. SNPs in the 3′UTR are frequent and have been proposed to contribute to individual variation in phenotype [10]. However, clear evidence for this concept was lacking. We show that SNPs in the 3′UTR of *KCNQ1* repress translation, and that these repressive SNPs largely determine the clinical severity in heterozygous carriers of an LQTS-linked *KCNQ1* mutation. When the suppressive SNPs reside on the normal *KCNQ1* allele, the normal allele is repressed and there is clear clinical manifestation of the mutation (long QTc durations and severe symptoms). When the suppressive SNPs reside on the mutant *KCNQ1* allele, this mutant allele is repressed and there are hardly any clinical manifestations of the mutation [3]. Our findings suggest that 3′UTR SNPs can alter microRNA binding and thereby modulate clinical expression of a disease-causing mutation. Our findings also suggest that in autosomal-dominant diseases, such as LQTS and BrS, disease severity can be modified by allelic imbalance induced by sequence variation in the 3′UTR stemming from the unaffected parent.

*A.S. Amin*

*Heart Failure Research Centre, Department of Clinical and Experimental Cardiology, Academic Medical Center, University of Amsterdam, Amsterdam, the Netherlands, e-mail: a.s.amin@amc.nl*

**References**

1. Amin AS, Tan HL, Wilde AAM. Cardiac ion channels in health and disease. Heart Rhythm. 2010;7:117–26.

2. Hofman N, Tan HL, Alders M, van Langen IM, Wilde AAM. Active cascade screening in primary inherited arrhythmia syndromes; does it lead to prophylactic treatment? J Am Coll Cardiol. 2010;55:2570–2576.

3. Amin AS, Giudicessi JR, Tijsen AJ, et al. Variants in the 3′untranslated region of the KCNQ1-encoded Kv7.1 potassium channel modify disease severity in patients with type 1 long QT syndrome in an allele-specific manner. Eur Heart J. 2012;33:714–23.

4. Amin AS, Meregalli PG, Bardai A, Wilde AAM, Tan HL. Fever increases the risk of cardiac arrest in a familial arrhythmia disease. Ann Intern Med. 2008;149:216–8.

5. Amin AS, De Groot EAA, Wilde AAM, Tan HL. Exercise-induced ECG changes in Brugada syndrome. Circ Arrhyth Electrophysiol. 2009;2:531–9.

6. Amin AS, Asghari-Roodsari A, Tan HL. Cardiac sodium channelopathies. Pflügers Archiv-Eur J Physiol. 2010;460:223–37.

7. Amin AS, Verkerk AO, Bhuiyan ZA, Wilde AAM, Tan HL. Novel Brugada syndrome causing mutation in ion-conducting pore of cardiac Na^+^ channel does not affect ion selectivity properties. Acta Physiologica. 2005;185:291–301.

8. Amin AS, Herfst LJ, Delisle BP, et al. Fever-induced QTc prolongation and ventricular arrhythmias in individuals with type 2 congenital long QT syndrome. J Clin Invest. 2008;118:2552–61.

9. Conne B, Stutz A, Vassalli JD. The 3′ untranslated region of messenger RNA: A molecular ‘hotspot’ for pathology? Nat Med. 2000;6:637–41.

10. Chen K, Rajewsky N. Natural selection on human microRNA binding sites inferred from SNP data. Nat Genet. 2006;38:1452–6.

**Cardiac resynchronisation therapy: determinants of patient outcome and emerging indications**

In this thesis, a comprehensive analysis was performed on the long-term effects of cardiac resynchronisation therapy (CRT) in selected patients with heart failure.

In the last decade, CRT has evolved as a very successful treatment strategy in selected patients with drug refractory heart failure. Evidence of large clinical trials undeniably established the beneficial effects of CRT in addition to optimal medical treatment on both morbidity and mortality. These effects include improved LV systolic function, improved clinical status, improved exercise tolerance and improved quality of life. The outcomes of these large trials resulted in the fact that for many years, CRT has been considered a class I indication in patients with end-stage heart failure (NYHA class III-IV) with an LVEF ≤35 % and a QRS complex duration ≥120 ms. Nonetheless, about 30 % of patients do not demonstrate response and it has become clear that many factors determine outcome in patients undergoing CRT. This thesis demonstrated that several patient characteristics have a strong influence on both response at 6-month follow-up and prognosis during long-term follow-up. Most important among these characteristics are gender, the presence of atrial fibrillation, renal failure and the presence of significant LV dyssynchrony. In addition to these patient characteristics, the position of the LV pacing lead in relation to the site of latest activation and potential scar tissue may have a great influence on outcome, especially in patients with ischaemic aetiology of heart failure. Integration of patient characteristics, LV lead position with information on LV dyssynchrony and scar tissue may help to improve patient selection and response to CRT.

**Fig 2:**
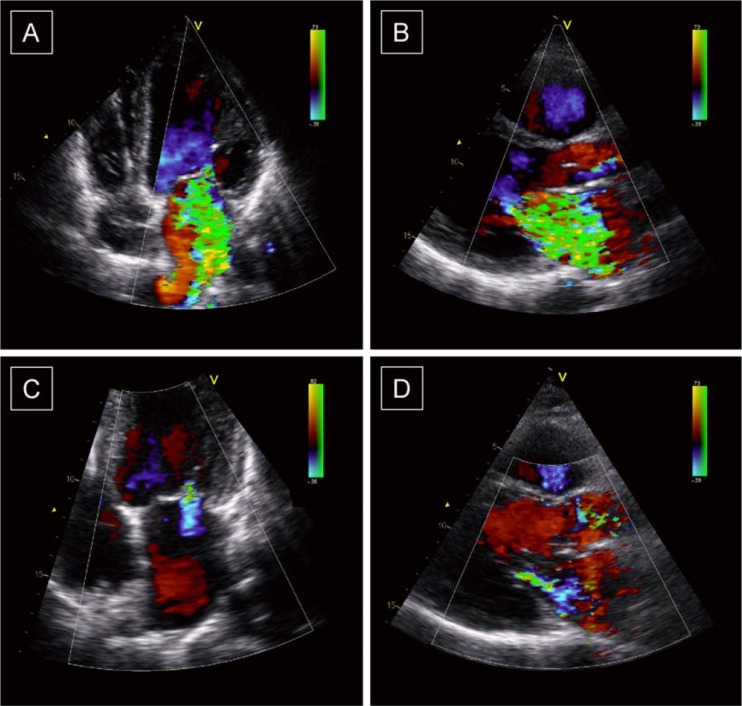
Example of a patient with a significant improvement in mitral regurgitation 6 months after cardiac resynchronization therapy. At baseline, the MR jet area exceeded 80 % of the left atrium area and the effective regurgitant orifice area (EROA) was 0.61 cm^2^ (Panels A [4-chamber view] and B [parasternal long-axis view]). At 6-month follow-up, the jet area decreased to 10 % of the left atrium area and the EROA was 0.19 cm^2^ (Panels C [4-chamber view] and D [parasternal long-axis view])

As large trials continued, more evidence for the benefit of CRT in other patient populations became available. As a result, since 2010 CRT has also been considered a class I indication in patients with mild symptoms of heart failure (NYHA class II) with an LVEF ≤35 % and a QRS complex duration ≥150 ms. It is not unlikely that the favourable effects of CRT will be extended to other patient groups in the coming years. These groups include asymptomatic (NYHA class I) patients, patients with a narrow QRS complex (<120 ms) or patients with heart failure but preserved LVEF (≥45 %). Many clinical trials are still underway and the future for CRT cannot be predicted at this moment. Data from this thesis showed that CRT also seems to improve other conditions frequently observed in patients with systolic heart failure. The improved LV systolic function induced by CRT increases cerebral blood flow (which is regularly decreased in heart failure patients). Furthermore, CRT-induced improvement in LV function also results in stabilisation of renal function, which is frequently decreased in heart failure patients. Finally, patients with severe functional MR and high operative risk also derive benefit from CRT. In these patients, CRT leads to a decrease in MR (Fig. 2), which in turn results in improved prognosis. Perhaps CRT may 1 day be used as an effective treatment strategy in these patient groups.

*R.J. van Bommel*

*Department of Cardiology, Leiden University Medical Center, Leiden, the Netherlands, e-mail: r.j.van_bommel@lumc.nl*

**The antithrombotic management of atrial fibrillation**

*A novel user-friendly score (HAS-BLED) to assess 1-year risk of major bleeding in patients with atrial fibrillation: the Euro Heart Survey*

Despite extensive use of oral anticoagulation (OAC) in patients with atrial fibrillation (AF) and the increased bleeding risk associated with such OAC use, no handy quantification tool for assessing this risk exists. We aimed to develop a practical risk score to estimate the 1-year risk for major bleeding (intracranial, hospitalisation, haemoglobin decrease > 2 g/L, and/or transfusion) in a cohort of real-world patients with AF. Based on 3978 patients in the Euro Heart Survey on AF with complete follow-up, all univariate bleeding risk factors in this cohort were used in a multivariate analysis along with historical bleeding risk factors. A new bleeding risk score termed HAS-BLED (Hypertension, Abnormal renal/liver function, Stroke, Bleeding history or predisposition, Labile international normalised ratio, Elderly (> 65 years), Drugs/alcohol concomitantly) was calculated, incorporating risk factors from the derivation cohort. Fifty-three (1.5 %) major bleeds occurred during 1-year follow-up. The annual bleeding rate increased with increasing risk factors. The predictive accuracy in the overall population using significant risk factors in the derivation cohort (C statistic 0.72) was consistent when applied in several subgroups. Application of the new bleeding risk score (HAS-BLED) gave similar C statistics except where patients were receiving antiplatelet agents alone or no antithrombotic therapy, with C statistics of 0.91 and 0.85, respectively. This simple, novel bleeding risk score (HAS-BLED) provides a practical tool to assess the individual bleeding risk of real-world patients with AF, potentially supporting clinical decision-making regarding antithrombotic therapy in patients with AF.

*Clinical correlates of immediate success and outcome at 1-year follow-up of real-world cardioversion of atrial fibrillation: the Euro Heart Survey*

In atrial fibrillation (AF) cardioversion is the cornerstone of the rhythm management strategy despite the lack of contemporary data on acute and long-term success. We aim to describe present-day cardioversion of AF and identify characteristics associated with immediate and long-term outcome. Based on the 5333 AF patients enrolled in the multicentre prospective Euro Heart Survey on AF we selected the 1801 patients undergoing cardioversion at enrolment. Sinus rhythm (SR) was restored in 630 of 712 (88 %), 458 of 643 (71 %), and 333 of 446 (75 %) (*P* < 0.001) of the electrical (ECV), intravenous (ivCCV), and oral (oCCV) chemical cardioversions, respectively, at the cost of few (4.2 %) major complications. In multivariate analysis, absence of chronic obstructive pulmonary disease (COPD) (*P* < 0.001), presence of paroxysmal AF (PAF) (*P* = 0.013), and use of biphasic waveform (*P* = 0.018) were predictors of successful ECV. For ivCCV PAF (*P* < 0.001), absence of valvular heart disease (*P* = 0.004), and heart failure (*P* = 0.009), the presence of hypertension (*P* = 0.018) and coronary artery disease (*P* = 0.007) were predictive. Success of oCCV was driven by PAF (*P* < 0.001) and a smaller left atrial dimension (*P* = 0.001). At 1-year follow-up 893 of 1271 (70 %) patients were in SR. Multivariate analysis revealed PAF (*P* < 0.001), shorter total AF history (*P* < 0.001), continuous use of Class Ic drugs or amiodarone during follow-up (*P* < 0.001), absence of COPD (*P* = 0.003), younger age (*P* = 0.004), and smaller left atrial dimension (*P* = 0.005) as independent predictors of SR at 1-year follow-up. Contemporary cardioversion of AF is routinely successfully and safely performed with a high proportion of patients in SR at 1-year follow-up.

*Potential net clinical benefit of population-wide implementation of apixaban and dabigatran among European patients with atrial fibrillation. A modelling analysis from the Euro Heart Survey*

Vitamin K antagonists (e.g. warfarin) are commonly underutilised, due to limitations such as the need for monitoring, in high-risk atrial fibrillation (AF) patients. We therefore aimed to model the potential impact on clinical outcomes in patients with AF with the use of the novel oral anticoagulant (OAC) drugs, apixaban and dabigatran. We identified all high-risk (CHA_2_DS_2_-VASc score ≥2) patients with non-valvular AF and known 1-year follow-up from the Euro Heart Survey on AF (EHS-AF). We modelled the expected numbers of clinical events on the novel OACs using published hazard ratios from their respective phase 3 clinical trials and calculated the numbers needed to treat and the mathematical net clinical benefit. Our analysis included 3400 patients [39 % females; mean (SD) age 67 (12) years; CHA_2_DS_2_-VASc score 3.0 (1.8)] of which 330 were excluded from the modelling analysis due to concomitant use of OAC and antiplatelet drugs. During 1-year follow-up, 108 (3.2 %) patients experienced thromboembolism, 51 (1.5 %) major bleeds and 146 (4.3 %) died. Compared with current treatments (i.e. warfarin, aspirin or nothing) the use of apixaban in high-risk patients would have potentially prevented an additional 17 deaths, 27 strokes and eight major bleeds within this cohort. With the use of dabigatran 150 mg twice daily (bid), 34 strokes could have been prevented and for dabigatran 110 mg bid, 16 strokes and six major bleeds would be avoided. Extrapolation of the data from the EHS-AF to the whole of Europe would translate into the prevention of an additional 64,573 major cardiovascular events and deaths each year among patients with a CHA_2_DS_2_-VASc ≥2, by the use of apixaban, 43,235 by the use of dabigatran 150 mg bid and 27,272 by the use of dabigatran 110 mg bid. In conclusion, based on this modelling exercise, the utilisation of apixaban and dabigatran for thrombo-prophylaxis could provide a profound annual mathematical net clinical benefit on stroke and major bleeds in European AF patients.
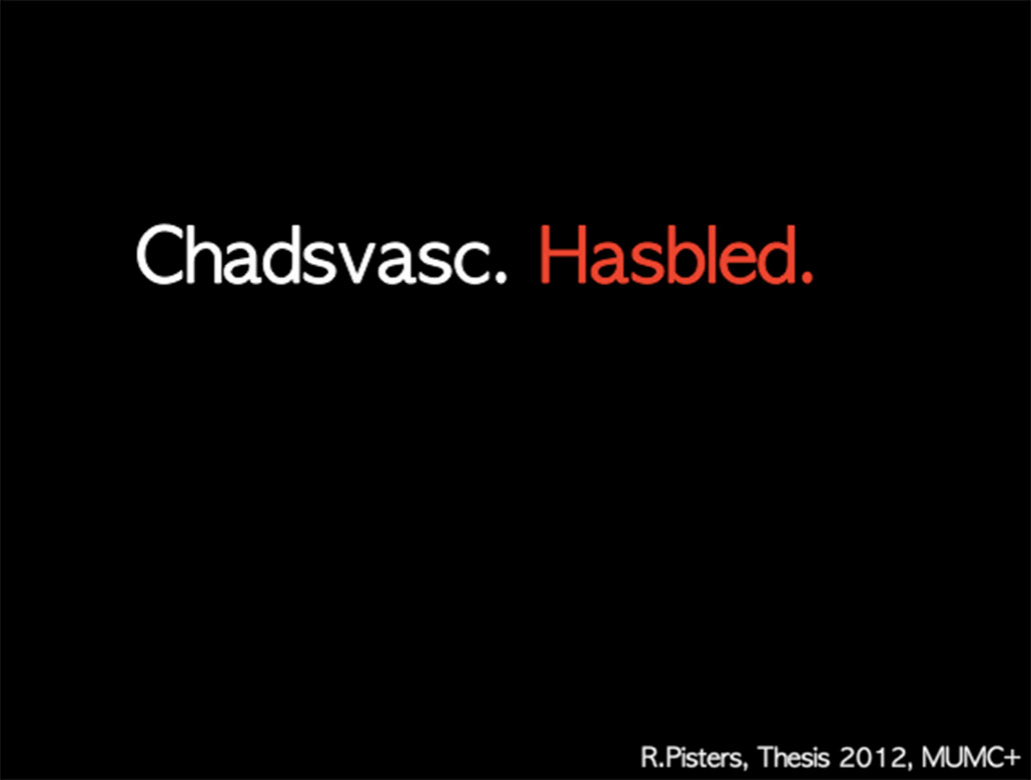


*R. Pisters*

*Department of Cardiology, Maastricht University Medical Centre, Maastricht, the Netherlands, e-mail: r.pisters@mumc.nl*

